# 3K3A-Activated Protein C Inhibits Choroidal Neovascularization Growth and Leakage and Reduces NLRP3 Inflammasome, IL-1β, and Inflammatory Cell Accumulation in the Retina

**DOI:** 10.3390/ijms241310642

**Published:** 2023-06-26

**Authors:** Yehonatan Weinberger, Ivan Budnik, Yael Nisgav, Dahlia Palevski, Gil Ben-David, José A. Fernández, Shany Nivinsky Margalit, Sarina Levy-Mendelovich, Gili Kenet, Dov Weinberger, John H. Griffin, Tami Livnat

**Affiliations:** 1 Rabin Medical Center, Ophthalmology Department and Laboratory of Eye Research Felsenstein Medical Research Center, Petah-Tikva 5251108, Israel; 2 Faculty of Medicine, Tel Aviv University, Tel-Aviv 6997801, Israel; 3 Department of Internal Medicine, The University of Iowa, Iowa City, IA 52242, USA; 4 Department of Molecular Medicine, The Scripps Research Institute, La Jolla, CA 92037, USA; 5 Sheba Medical Center, The Amalia Biron Thrombosis and Hemostasis Research Institute, Tel-Hashomer 52621, Israel

**Keywords:** activated protein C, choroidal neovascularization, inflammation NLRP3, microglia

## Abstract

3K3A-Activated Protein C (APC) is a recombinant variant of the physiological anticoagulant APC with cytoprotective properties and reduced bleeding risks. We studied the potential use of 3K3A-APC as a multi-target therapeutic option for choroidal neovascularization (CNV), a common cause of vision loss in age-related macular degeneration. CNV was induced by laser photocoagulation in a murine model, and 3K3A-APC was intravitreally injected. The impact of 3K3A-APC treatment on myeloid and microglia cell activation and recruitment and on NLRP3 inflammasome, IL-1β, and VEGF levels was assessed using cryosection, retinal flat-mount immunohistochemistry and vascular imaging. Additionally, we evaluated the use of fluorescein angiography as a surrogate marker for in vivo evaluation of the efficacy of 3K3A-APC treatment against leaking CNV lesions. Our results demonstrated that 3K3A-APC treatment significantly reduced the accumulation and activation of myeloid cells and microglia in the CNV area and decreased the NLRP3 and IL-1β levels at the CNV site and the surrounding retina. Furthermore, 3K3A-APC treatment resulted in leakage regression and CNV growth suppression. These findings indicate that the anti-inflammatory activities of 3K3A-APC contribute to CNV inhibition. Our study suggests the potential use of 3K3A-APC as a novel multi-target treatment for CNV.

## 1. Introduction

Choroidal neovascularization (CNV) is characterized by the growth of newly formed abnormal, fragile, and leaky blood vessels that emerge from the choroid and extend through the Bruch’s membrane and the retinal pigment epithelium (RPE) to the subretinal space or into the neurosensory retina, leading to exudation and hemorrhage and ultimately to vision loss [1]. CNV is a common cause of visual impairment in neovascular age-related macular degeneration (nAMD) patients. Blood-retina barrier (BRB) disruption, accompanied by leakage and neuroinflammation, exacerbates the progression of nAMD and vision loss [1,2,3]. The current efficacious treatments for nAMD are mainly based on anti-vascular endothelial growth factor (VEGF) agents. However, suboptimal response, slow loss of efficacy, and long-term concerns regarding the continuity of the neurodegenerative process hinder their complete success [4,5]. The development of multi-target treatments holds promise in effectively targeting the pivotal factors underlying CNV, thereby halting the progression of neurodegeneration and improving visual outcomes.

Strong clinical and experimental evidence links the innate immune responses to the pathophysiology of CNV and nAMD [3,6]. Inflammatory mediators, such as components of the complement system, cytokines, and chemokines, and accumulation of microglia and macrophages in the CNV area amplify the pathological neovascularization associated with the disease [3,6,7]. One key pro-inflammatory pathway implicated in promoting nAMD involves the activation of the NACHT, LRR, and PYD domains-containing protein 3 (NLRP3) inflammasome, which is thought to contribute to tissue damage [8,9]. However, the underlying mechanisms linking inflammation and CNV formation are multifactorial and complex and have not yet been fully elucidated.

Activated protein C (APC) is a physiological anticoagulant derived from its zymogen protein C. Apart from its anticoagulant activity in the plasma, APC has cellular activities mediated mainly by its cellular receptors, endothelial protein C receptor (EPCR), protease-activated receptor (PAR)1, and PAR3 [10,11,12]. The well-established anti-inflammatory, anti-apoptotic, and blood-barrier-protective activities of APC [10,11] have led to the approval of recombinant wild-type APC (wt-APC) as a treatment for adult patients with severe sepsis [13]. However, therapy was complicated by bleeding events that were considered side effects of APCs’ anticoagulant properties [13]. By replacing three lysine residues (KKK191–193) with three alanine residues, Mosnier et al. engineered the APC analog 3K3A-APC [14]. Replacing the three residues reduces APC’s interactions with the clotting factor Va and diminishes its anticoagulant activity [14]. Although 3K3A-APC has markedly reduced anticoagulant activity, it sustains APCs’ pleiotropic cytoprotective activities and preserves the interactions with its cell receptors, including binding to EPCR and PARs [11,14].

Various models of neurological injuries have shown that 3K3A-APC has neuroprotective effects manifesting as protection of blood-brain barrier (BBB) function, inhibition of neuroinflammation, inhibition of neuronal apoptosis, and regenerative effects targeting neuronal stem cells. These highlight the pleiotropic, cytoprotective functions of 3K3A-APC in the brain, central nervous system (CNS), and neurodegenerative diseases [11,15,16]. Following optimization of the recombinant 3K3A-APC for human therapeutic use, a multicenter randomized phase 2 RHAPSODY trial successfully established the safety of 3K3A-APC as a potential treatment for severe ischemic stroke [17].

To our knowledge, our group is the only one studying the effects of 3K3A-APC in the retina [18,19]. We previously showed that intravitreal (IVT) treatment with 3K3A-APC inhibits CNV growth and significantly reduces VEGF levels at CNV sites [18]. Recently we demonstrated the anti-inflammatory activities of 3K3A-APC in the inflamed retina using an endotoxin-induced uveitis (EIU) murine model [19]. We showed that 3K3A-APC treatment restricted inflammatory cell infiltration from the inner blood vessels of the retina into the retinal parenchyma and inhibited microglia accumulation and activation. Moreover, 3K3A-APC inhibited inflammasome activation in the retina, as reflected by decreased NLRP3 and IL-1β levels [19].

In this study, we aimed to investigate the potential clinical applicability of 3K3A-APC as a multi-target therapeutic option for CNV, considering the limited efficacy of single-action-single-target agents in addressing the complex pathophysiology behind CNV.

We used the laser-induced CNV murine model to evaluate the anti-inflammatory properties of 3K3A-APC. In this model, laser photocoagulation perforates the Bruch’s membrane-RPE layers, initiating the growth of abnormal blood vessels from the choroid. These vessels penetrate the RPE and extend into the sub-retinal space [20]. Though this model represents an acute injury rather than the progressive, age-dependent development of CNV observed in human nAMD, it is widely recognized as a valuable tool for investigating CNV pathogenesis and exploring potential treatments [21]. Notably, in our previous work, we demonstrated the anti-inflammatory effects of 3K3A-APC in an acute inflammation model induced via endotoxin administration to the vitreous [19]. In the current model, inflammation occurs concurrently with the trigger of CNV.

Our results demonstrate the effect of 3K3A-APC on myeloid and microglial cell accumulation and activation in the CNV area, as well as its impact on NLRP3 inflammasome activation. Furthermore, as effective management of sub-RPE, sub-retinal, and intra-retinal leakage associated with CNV is critical in preventing further damage and vision loss, we conducted in vivo monitoring of the impact of 3K3A-APC treatment on CNV leakage using fluorescein angiography (FA). Our findings provide additional evidence supporting the promising clinical utility of 3K3A-APC as a pleiotropic therapeutic agent for CNV.

## 2. Results

### 2.1. 3K3A-APC -APC Reduces NLRP3 Levels at the CNV Lesion

**Figure 1:**
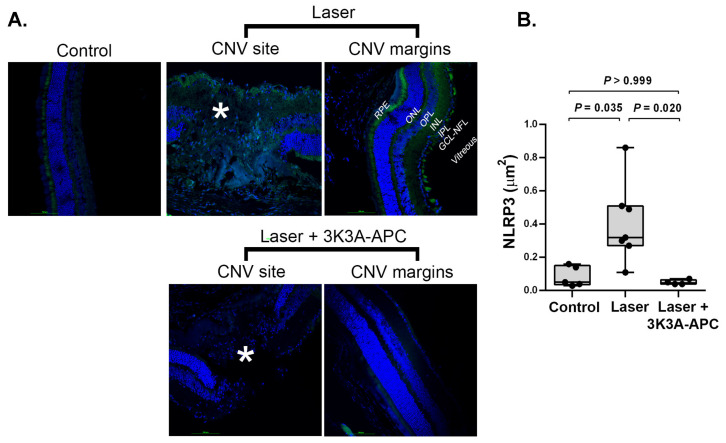
Representative figures of retinal cryosections immunostained with NLRP3 antibodies are shown in panel (**A**). Blue represents cell nuclei; green represents NLRP3 (scale bar = 100 µm). In control eyes, without any intervention, minimal NLRP3 expression was detected, mostly restricted to the outer part of the retina, reflecting the constitutive expression of NLRP3 inflammasome in the retina (left panel). In the eyes subjected to lasers and injected with saline, NLRP3 staining was elevated at the CNV site (marked with an asterisk) and extended to the lesion margins (Laser panels). Treatment with 3K3A-APC resulted in significant attenuation of NLRP3 staining, with minimal staining observed at the CNV site (marked with an asterisk) and the surrounding retina (Laser + 3K3A-APC panels). Quantitative analysis of the NLRP3 area (µm^2^) confirmed the statistical significance of these findings, as illustrated in panel (**B**). Positive staining areas were calculated using values from 4 microscopic fields (2 fields/slides × 2 slides/animal), which were averaged and used as raw data for further analysis. Results are presented as box-and-whiskers plots. The boxes span the 25th to the 75th percentile, the line inside each box denotes the median, and the whiskers span the lowest to the highest observations. Comparisons were performed with the Kruskal–Wallis test followed by Dunn’s post hoc test (n = 5–6 per group). CNV—Choroidal neovascularization; GCL—ganglion cell layer; INL—inner nuclear layer; IPL—inner plexiform layer; ITV—intravitreal; NFL—nerve fiber layer; ONL—outer nuclear layer; OPL—outer plexiform layer; RPE—retinal pigment epithelium; *—lesion area. NLRP3—NLR family pyrin domain containing 3.

The NLRP3 inflammasome, a crucial regulator of the inflammatory response, has been implicated in the pathogenesis of CNV [8,9]. We assessed the impact of 3K3A-APC treatment on NLRP3 levels at the CNV sites. One hour after CNV induction, either 3K3A-APC or saline was intravitreally injected, and four days later, retinal cryosections were stained for NLRP3. Representative images of retinal cryosections immunostained with NLRP3 antibodies (green) are shown in Figure 1A. Minimal staining of NLRP3 was detected in control eyes that were not subjected to lasers. NLRP3 staining was mostly restricted to the outer part of the retina, with very little observed in the outer and inner plexiform layers (OPL and IPL, respectively). This staining pattern reflects the constitutive expression of NLRP3 inflammasome in various cell types, including the RPE, retinal microglia, Müller cells, and astrocytes [22]. In eyes subjected to lasers and treated with saline, prominent staining for NLRP3 was observed in the CNV area, extending to the RPE and throughout all retinal neurosensory layers, including the OPL and IPL. Notably, NLRP3 expression was not only limited to the CNV site itself but also extended to the margins of the CNV lesion. In contrast, eyes subjected to lasers and treated with 3K3A-APC showed a significant attenuation of NLRP3 staining, with minimal staining observed at the CNV site (marked with an asterisk), resembling the staining pattern of control eyes without CNV. Quantitative analysis of NLRP3 areas confirmed the statistical significance of these findings, as illustrated in Figure 1B. The increase in NLRP3 levels observed following CNV induction was effectively prevented by 3K3A-APC treatment (median 0.32 [IQR 0.27–0.51] μm^2^ vs. 0.05 [IQR 0.04–0.07] μm^2^, respectively, *p* = 0.020), reaching levels comparable to those observed in control eyes (median 0.05 [IQR 0.04–0.15] μm^2^, *p* > 0.999).

### 2.2. 3K3A-APC Reduces IL-1β Levels at the CNV Lesion

**Figure 2:**
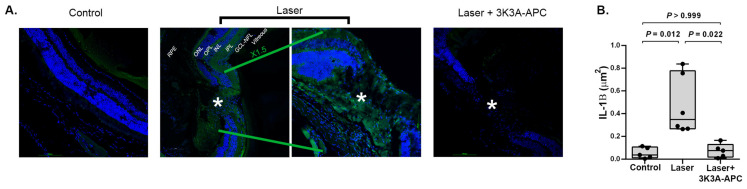
Representative figures of retinal cryosections immunostained with IL-1β antibodies are shown in panel (**A**). Blue represents cell nuclei; green represents NLRP3 (scale bar = 100 µm). In control eyes without any intervention, minimal IL-1β expression was detected (left panel). IL-1β levels increased significantly after CNV induction, observed at the CNV site (marked with an asterisk) and extended to the lesion margins (middle panels). Treatment with 3K3A-APC significantly attenuated IL-1β staining, with minimal staining observed at the CNV site (marked with an asterisk) and the surrounding retina (right panel). Quantitative analysis of IL-1β area (µm^2^) confirmed the statistical significance of these findings, as illustrated in panel (**B**). Positive staining areas were calculated using values from 4 microscopic fields (2 fields/slides × 2 slides/animal), which were averaged and used as raw data for further analysis. Results are presented as box-and-whiskers plots. The boxes span the 25th to the 75th percentile, the line inside each box denotes the median, and the whiskers span the lowest to the highest observations. Comparisons were performed with the Kruskal–Wallis test followed by Dunn’s post hoc test (n = 5–6 per group). CNV—Choroidal neovascularization; GCL—ganglion cell layer; INL—inner nuclear layer; IPL—inner plexiform layer; ITV—intravitreal; NFL—nerve fiber layer; ONL—outer nuclear layer; OPL—outer plexiform layer; RPE—retinal pigment epithelium; *—lesion area. NLRP3—NLR family pyrin domain containing 3.

The NLRP3 inflammasome activation ultimately results in the release of the pro-inflammatory cytokines interleukin (IL)-1β and IL-18 [8,23]. To evaluate the ability of 3K3A-APC to inhibit inflammasome activation, IL-1β levels were measured at the CNV sites. One hour after CNV induction, either 3K3A-APC or saline was intravitreally injected, and four days later, retinal cryosections were prepared and stained for IL-1β. Representative figures of retinal cryosections immunostained with IL-1β antibodies (green) are shown in Figure 2A. In control eyes, minimal IL-1β staining was observed. However, in eyes with CNV treated with saline, a pronounced elevation in IL-1β staining was observed at the CNV site (marked with an asterisk), at the RPE and all neurosensory retinal layers, including the OPL and IPL. In contrast, eyes with CNV treated with 3K3A-APC showed almost no IL-1β staining in the CNV area or throughout the retina layers, indicating a significant reduction in IL-1β levels with 3K3A-APC treatment. Quantitative analysis showed a statistically significant elevation of IL-1β following CNV induction that was dramatically reduced by 3K3A-APC treatment (median 0.35 [IQR 0.27–0.78] μm^2^ vs. 0.08 [IQR 0.02–0.13] μm^2^, respectively, *p* = 0.022), reaching levels comparable to those observed in control eyes (median 0.04 [IQR 0.01–0.11] μm^2^, *p* > 0.999, Figure 2B).

### 2.3. 3K3A-APC Treatment Reduces Microglia Recruitment and Activation at the CNV Lesion

Microglia, the immune cells residing in the retina, are well-known to express IBA1 (ionized calcium-binding adaptor molecule 1), which serves as a reliable marker for microglial activation [24]. Under normal physiological conditions, microglia exhibit a quiescent state and display a ramified morphology. However, in response to injury, microglial cells undergo activation and adopt an amoeboid shape [7,25,26]. We assessed the presence of IBA1+ cells at the sites of CNV and determined their activation state based on their morphology, characterized as either activated (amoeboid shape) or non-activated (ramified shape).

**Figure 3:**
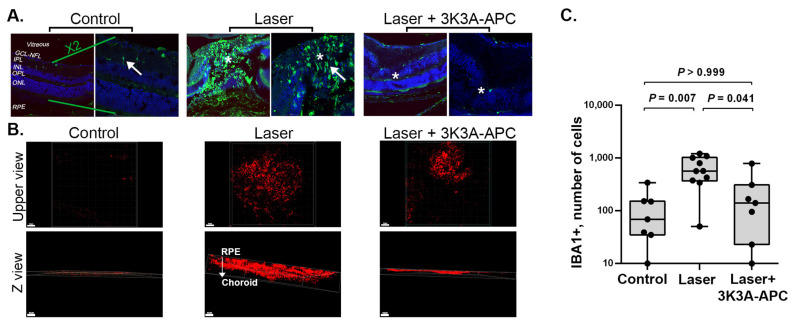
Representative images of retinal cryosections immunostained with IBA1 antibodies, a specific marker for microglia (green), and DAPI (blue) as a nuclei marker, are shown in panel (**A**). The right panel of each group represents a higher magnification (scale bar = 50 µm) of the left panel (scale bar = 100 µm). Control eyes without any intervention showed scant and ramified (non-active, marked by an arrow) IBA1+ cells, mainly in the inner retinal layers (left panel). In the untreated eyes with CNV, a significant accumulation of amoeboid, active (marked with by an arrow) microglial cells was observed in the CNV area and the surrounding retina (middle panel). However, 3K3A-APC treatment dramatically reduced microglial accumulation (right panel). IBA1+ cells and their response to 3K3A-APC treatment were further assessed in RPE-choroid flat-mounts. Intravitreal injections of either 3K3A-APC or saline were administered one hour after CNV induction. Panel (**B**) displays representative upper-view (upper panels) and depth Z-view (lower panels) color images of RPE-choroid flat-mounts stained with IBA1 antibodies (red) and scanned from the RPE into the choroid. Minimal staining of IBA1+ cells was detected in control eyes not subjected to laser applications (control panels). However, deeper and more extensive penetration of IBA1+ cells was observed from the RPE surface throughout the depth of the choroid in eyes exposed to lasers and treated with saline (laser panels). Treatment with 3K3A-APC significantly reduced the number and penetration of IBA1+ cells into CNV sites, and most of them were located at the edge of the RPE and not deeper into the choroid, as shown by the Z view (laser 3K3A-APC panels). Quantitative analysis of IBA1+ cells area (µm^2^) confirmed the statistical significance of these findings (panel (**C**)). Results are presented as box-and-whiskers plots. The boxes span the 25th to the 75th percentile, the line inside each box denotes the median, and the whiskers span the lowest to the highest observations. Comparisons were performed with the Kruskal–Wallis test followed by Dunn’s post hoc test (n = 7–10 per group). CNV—Choroidal neovascularization; INL—inner nuclear layer; IPL—inner plexiform layer; NFL—nerve fiber layer; ONL—outer nuclear layer; OPL—outer plexiform layer. IBA1—ionized calcium-binding adaptor molecule 1; RPE—retinal pigment epithelium; *—lesion area.

One hour after CNV induction, either 3K3A-APC or saline was injected intravitreally. Four days later, retinal cryosections were prepared and stained for IBA1 (Figure 3A). Control eyes, without any intervention, showed scant and ramified (non-active, example marked by an arrow) IBA1+ cells, mainly in the inner retinal layers. In the untreated eyes with CNV, a significant increase in microglial cells was observed in the CNV area and the surrounding retina (middle panel). Furthermore, these cells underwent a morphological change towards an amoeboid shape, indicating their activation (example marked by an arrow). However, in eyes treated with 3K3A-APC after laser induction, both a reduction in the number of microglial cells and inhibition of their activation, indicated by ramified morphology, were detected (Figure 3A, right panel).

We further assessed the presence of IBA1+ cells and their response to 3K3A-APC in RPE-choroid flat-mounts. Intravitreal injections of either 3K3A-APC or saline were administered one hour after CNV induction. After one week, RPE-choroid specimens were prepared as flat-mounts and stained with anti-IBA1 antibodies. The specimens were positioned with the RPE layer facing upwards and the choroid resting on the slide and were scanned using confocal microscopy from the RPE into the choroid. Figure 3B shows representative upper-view (upper panels) and depth Z-plane (lower panels) color images.

Minimal staining of IBA1+ cells was detected in control eyes not subjected to laser applications. However, in eyes exposed to lasers and treated with saline, deeper and more extensive accumulation of IBA1+ cells was observed from the RPE surface throughout the depth of the choroid. Treatment with 3K3A-APC significantly reduced the accumulation of IBA1+ cells in CNV sites. Notably, similar to our observation in the cryosection, the IBA1+ cells were located at the edge of the RPE rather than deeper in the choroid.

Quantitative measurements of IBA1+ cell counts in the entire RPE-choroid specimen (Figure 3C) showed a significant reduction in the median count of IBA1+ cells in the 3K3A-APC-treated eyes compared to the untreated eyes (141 [IQR 23–311] vs. 569 [IQR 367–1032] cells, *p*=0.041, respectively). These findings indicate that 3K3A-APC treatment restricted the accumulation and activation of microglia cells within the site of the CNV lesion.

### 2.4. 3K3A-APC—Treatment Suppresses Myeloid Cell Accumulation and CNV Growth

**Figure 4:**
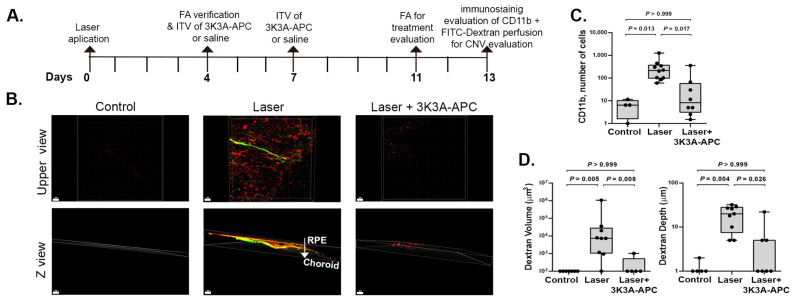
The timeline of the experiment conducted to evaluate the efficacy of 3K3A-APC treatment on myeloid cell accumulation and CNV suppression is presented in panel (**A**). RPE-choroid specimens were positioned with the RPE layer facing upwards and the choroid resting on the slide. Retinal blood vessels were stained using fluorescein isothiocyanate (FITC)-dextran perfusion (green), and myeloid cells were stained using anti-CD11b antibody (red), and scanned using confocal microscopy from the RPE into the choroid. Panel (**B**) displays representative upper-view (upper panels) and depth Z-view (lower panels) color images. Panel (**C**): Quantification of the total number of CD11b+ cells within the RPE-choroid specimens. 3K3A-APC treatment significantly reduces the total CD11b+ cells in the CNV area. Panel (**D**): Quantification of CNV volume (µm^3^) and penetration depth of blood vessels (µm) indicates that 3K3A-APC treatment suppresses CNV growth and reduces penetration. Results are presented as box-and-whiskers plots. The boxes span the 25th to the 75th percentile, the line inside each box denotes the median, and the whiskers span the lowest to the highest observations. Comparisons were performed with the Kruskal–Wallis test followed by Dunn’s post hoc test *(n* = 5–9 eyes per group). CNV—Choroidal neovascularization; FA—fluorescein angiography; RPE—retinal pigment epithelium.

Next, we aimed to investigate the effect of 3K3A-APC treatment on myeloid cell accumulation when administered after established CNV-related inflammation is already present. As we observed robust inflammation on days 4–7 after CNV induction (Figure 1, Figure 2 and Figure 3), we used a treatment regimen of 2 consecutive intravitreal injections of 1 µg/µL/eye 3K3A-APC, administered on days 4 and 7 post-CNV induction. The concentration of 3K3A-APC was chosen based on previously performed studies [18,19]. Then, 7 days after the last treatment (on day 13), an in-vivo injection of fluorescein isothiocyanate (FITC)-dextran was performed to mark retinal blood vessels fluorescently. Immediately after this, RPE-choroid specimens were prepared as flat-mounts, stained with the myeloid marker CD11b, and scanned using confocal microscopy. The timeline of the experiment is present is presented in panel 4A. Figure 4B shows representative upper view and depth Z-plane color images of CD11b+ cells in RPE-choroid flat-mounts and the CNV vascular perfusion. In eyes not subjected to any intervention, no presence of CD11b+ cells or CNV’s vascular component was found. Eyes subjected to CNV induction with saline treatment showed a significant presence of CD11b+ cells throughout the CNV depth, which reduced significantly with 3K3A-APC treatment. Quantification of CD11b+ cells in the RPE-choroid specimens is depicted in Figure 4C. As expected, almost no CD11b+ staining existed in eyes not subjected to CNV induction (median of 7 [IQR 2–10]). Eyes with laser-induced CNV and vehicle treatment showed markedly elevated CD11b+ cell numbers in the tissue (median of 215 [IQR 98–370] cells). 3K3A-APC treatment dramatically reduced CD11b+ cell count (median 8 [IQR 3–58] cells, *p* = 0.017), similar to eyes not subjected to lasers (*p* > 0.999). The total volume of CNV was significantly regressed by 3K3A-APC treatment. The median volume of CNV was 7342 µm^3^ (IQR 1054–27904) in saline-treated eyes, whereas it was 0 µm^3^ (IQR 0–513) in 3K3A-APC-treated eyes (*p* = 0.008). Similarly, the median depth of CNV was 20 µm (IQR 7.5–28) in saline-treated eyes, compared to 0 µm (IQR 0–5) in 3K3A-APC-treated eyes (*p* = 0.026), as shown in Figure 4D. These results corroborate our earlier findings on the ability of 3K3A-APC to suppress CNV growth and penetration from the choroid into the retina [18] and demonstrate that treatment with 3K3A-APC reduces the involvement of inflammatory cells in the neovascular process.

### 2.5. 3K3A-APC Treatment Reduces Leakage from CNV with a Correlation to Decreased VEGF Levels

**Figure 5:**
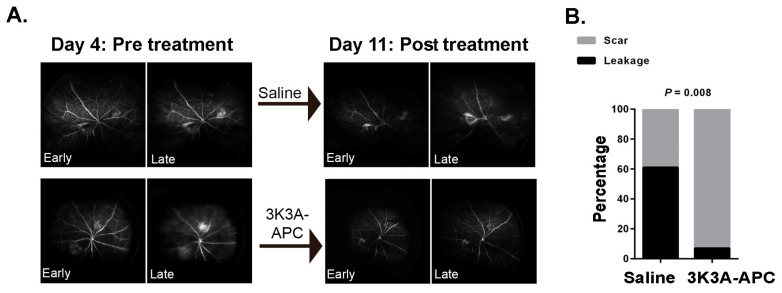
Pre-treatment fluorescein angiography (FA) was conducted on day 4 post-laser to confirm the presence of leakage from CNV. Mice with confirmed leakage were then treated with 3K3A-APC or saline, and additional FA evaluated treatment efficacy on day 11 post-laser. Panel (**A**) shows representative dynamic FA images of the same eye, taken on days 4 and 11, which masked retina specialists used to evaluate leakage from CNV. A quantitative assessment of leaking lesions, comparing the percentage of leaking lesions in 3K3A-APC or vehicle-treated eyes, performed on day 11, is presented in panel (**B**). To account for 2 lesions per mouse, the data were analyzed by using a generalized estimating equation, with the lesion status (“leaking” or “non-leaking”) as a binary outcome and 3K3A-APC treatment (“no” or “yes”) as a between-subjects factor. The resulting *p*-value is presented in the graph. CNV—Choroidal neovascularization.

In clinical scenarios, such as nAMD, increased permeability of the neovessels can result in excessive fluid accumulation and hemorrhage into the surrounding tissues, ultimately leading to vision loss. In our next step, we aimed to simulate these scenarios, evaluate the protective role of 3K3A-APC against leaking CNV lesions, and determine whether leakage on FA can serve as a surrogate marker for in-vivo evaluation of the efficacy of 3K3A-APC treatment. To achieve this, we first induced CNV growth through laser photocoagulation. We conducted pre-treatment FA on day 4 to confirm the presence of leakage from CNV, as can be detected in cases in which FA is performed in humans. Mice with confirmed leakage were then divided into 2 groups: 3K3A-APC or saline treatment. The chosen treatment regimen was based on the consecutive injections applied in pre-clinical trials of 3K3A-APC [16,27] and the common practice with anti-VEGF injections. Therefore, we used a treatment regimen of 2 consecutive intravitreal injections of 1 µg/µL/eye 3K3A-APC, administered on day 4 and day 7 post-CNV induction. Then, 11 days post-laser, we performed additional FA to assess the efficacy of 3K3A-APC treatment on CNV leakage. The experiment timeline is depicted in Figure 4A. Figure 5A displays representative FA images of 3K3A-APC and saline-treated eyes that masked retina specialists used to evaluate leakage from CNV. On day 4, before treatment, dynamic FA imaging showed hyperfluorescence at lesion sites in both arms on early images, which intensified with the blurring of margins on late images, indicating active, leaking CNV lesions. On day 11, imaging of saline-treated eyes showed active leakage from CNV. In contrast, 3K3A-APC-treated eyes showed scant early hyperfluorescence that was stable without intensified hyperfluorescence and no blurring of margins, indicating non-active (or scarred) lesions. A quantitative assessment comparing the percentage of leaking lesions in 3K3A-APC or saline-treated eyes, performed on day 11, is presented in Figure 5B. Pathologically significant leakage was present in 11 out of 18 CNV lesions in saline-injected mice but only in 1 out of 14 CNV lesions in 3K3A-APC-treated mice (61% vs. 7%). The analysis showed that the odds of forming non-leaking lesions increase by 20.43 (95% CI 2.19–190.50, *p* = 0.008) if 3K3A-APC is injected (Table S1). Moreover, a statistical analysis of the relationship between the leakage status of each specimen and its VEGF levels (quantified by VEGF immunostaining in retinal flat-mounts) showed that the mean VEGF volume in non-leaking lesions was 4607 µm^3^ vs. 131,529 µm^3^ in leaking lesions (*p* = 0.009, Table S2). These findings indicate a strong correlation between leakage and elevated VEGF levels.

## 3. Discussion

CNV is a common cause of vision impairment in patients with nAMD [1,2]. Moreover, CNV can manifest as a secondary pathology of inherited and acquired conditions, including high myopia, angioid streaks, and hereditary, traumatic, or inflammatory disorders [28]. Despite the effectiveness of anti-VEGF-based treatments, it is necessary to consider approaches that can inhibit the continuity of the neurodegenerative process and serve as an alternative for unresponsive patients [4,5]. Given the multifactorial aspects that contribute to CNV development, a multi-target approach that simultaneously addresses multiple pathogenic mechanisms may offer an effective strategy to achieve optimal therapeutic outcomes, which have not been fully achieved by solely targeting VEGF [29]. Therefore, there is a substantial effort to identify potential neuroprotective agents. This includes not only pharmacological approaches, but also non-pharmacological interventions such as sub-threshold diode micropulse laser therapy, which show promise due to their potential to induce retinal neuroprotection [30]. Our group has demonstrated that treatment with wt-APC effectively inhibits CNV development. Its protective effects in the retina are partially mediated through VEGF reduction and signaling via the Tie2 receptor [18,31]. Moreover, a small clinical trial conducted by Hara et al. on the long-term safety of intravitreal administration of plasma-derived wt-APC for treating central retinal vein occlusion (CRVO) [32] raises the possibility of considering the use of wt-APC for clinical applications in treating CNV. However, using wt-APC as a treatment for fragile and abnormal blood vessels associated with CNV may not be suitable due to its anticoagulant properties, which can increase the risk of ocular hemorrhage. 3K3A-APC, a recombinant engineered APC variant with reduced anticoagulant activity [14], sustains wt-APC’s cellular cytoprotective activities, including anti-inflammatory activities, blood barrier stabilization, and anti-apoptotic and neuroprotective effects [10,11]. In a comparative study using a murine model of CNV, we demonstrated that 3K3A-APC maintains wt-APC’s beneficial activities in the retina, as evidenced by the regression of CNV and reduced VEGF levels at the site of CNV [18].

The anti-inflammatory effects of wt-APC and 3K3A-APC have been widely recognized [10,11,12,33,34,35,36]. Recently, we demonstrated the anti-inflammatory activities of 3K3A-APC in the retina using an acute model of retinal inflammation induced by intraocular administration of LPS in mice eyes. [19]. Since inflammation is known to play a role in the pathogenesis of CNV development [3,23,25], we hypothesize that the anti-inflammatory activities of 3K3A-APC may contribute to CNV regression and could offer a potentially promising safe approach for treating CNV.

The inflammasome, a pro-inflammatory protein, plays a crucial role in the innate immune response by sensing danger signals. The NLRP3 inflammasome is expressed in various eye parts, including the RPE, retinal microglia, Müller cells, astrocytes, conjunctiva, trabecular meshwork, and corneal epithelial cells [22]. Assembly and activation of the NLRP3 inflammasome lead to the caspase-1-dependent release of pro-inflammatory cytokines, IL-1β, and IL-18 [8,23]. Inflammasome activation contributes to tissue damage in ocular diseases, including glaucoma, diabetic retinopathy, and AMD [1,9]. Our findings indicate that treatment with 3K3A-APC effectively reduces the levels of NLRP3 and its downstream effector, IL-1β, in the CNV area and its surrounding regions following laser-induced CNV development (Figure 1 and Figure 2). This suggests that 3K3A-APC may act by preventing the assembly and activation of the NLRP3 inflammasome. Our observation is consistent with previous research by Nazir et al., which demonstrated that APC could attenuate inflammasome activity by reducing NLRP3 expression and activating caspase-1 and IL-1β, independently of its anticoagulant properties but dependent on signaling via PAR-1, in murine models of myocardial and renal ischemia-reperfusion injury [37]. Healy et al. also showed that APC could reduce inflammasome activity in stimulated human monocytes, as evidenced by decreased caspase-1 activity and IL-1β release [35]. Furthermore, Yan et al. reported that 3K3A-APC could ameliorate early brain injury induced by subarachnoid hemorrhage by suppressing pyroptosis through inhibition of the NLRP3 inflammasome and reduction in IL-1β levels [36]. In line with these findings, our recent study demonstrated that treatment with 3K3A-APC effectively restricts NLRP3 inflammasome activation and reduces IL-1β in LPS-induced retinal inflammation [19]. Much attention has been paid to the role of NLRP3 inflammasome activation in RPE cells. However, recent findings shift the focus and provide evidence that inflammasome activation occurs predominantly in activated macrophages and retinal microglia cells that infiltrate the site of CNV lesions [8]. We observed a significant increase in NLRP3 levels at the CNV lesions, which may suggest microglia as the source of NLRP3, but also in the RPE and the ganglion cell-nerve fiber layer (Figure 1). It is still unclear which specific cell types in the retina 3K3A-APC acts on to limit inflammasome activation, making it an important open question that needs to be addressed.

Microglia, the resident immune cells of the retina, are located in the nerve fiber layer, inner plexiform layer, and outer plexiform layer of the healthy eye, where they are closely associated with the vasculature, neuronal synapses, and glia [3,24,26,38]. As others have shown [7,25,26], we observed an accumulation of amoeboid active microglia in the site of CNV throughout the depth of the RPE-choroid. A significant reduction in microglial accumulation and a shift towards a more quiescent, ramified morphology were noted upon 3K3A-APC treatment. These findings are consistent with recent research demonstrating the inhibitory effects of 3K3A-APC on microglial accumulation and activation in the inflamed retina [19]. Additionally, our results align with previous studies using preclinical models of neurological injuries, such as ischemic injury of the corpus callosum, Alzheimer’s disease, and multiple sclerosis (MS) [16,27,33,39], which have shown that 3K3A-APC treatment can effectively inhibit microglial migration and activation.Furthermore, the increase in inflammatory cells in the CNV area can be attributed to the infiltration of circulating CD11b+ monocytes positive for various proangiogenic factors and inflammatory cytokines [38]. Subhi et al. suggested that the proportion of CD11b+ circulating monocytes in patients with nAMD could provide valuable prognostic information regarding the long-term need for intravitreal therapy [40]. We found that 3K3A-APC treatment effectively reduced the accumulation of CD11b+ cells in the CNV area, even when administered during extensive inflammation (Figure 4). The ability of 3K3A-APC to inhibit myeloid cell extravasation of CD11b+ circulating monocytes out of retinal blood vessels into the retinal parenchyma [19], along with its well-established endothelial blood barrier stabilization activities [10,11,41], can explain our observation. However, due to a lack of specific markers, differentiating microglia from infiltrating mononuclear phagocytes is difficult. Notably, while the laser-induced CNV model does resemble human CNV progression, it simulates an acute, trauma-induced inflammatory process accompanied by significant neuroretinal damage. This differs from the chronic, age-related degeneration typically observed in human CNV. Therefore, caution is necessary when extrapolating our findings, particularly those concerning inflammation related to nAMD. Despite these inherent limitations, the laser-induced CNV model remains an invaluable resource for investigating CNV mechanisms and evaluating potential treatments [21].

Given the high prevalence of leakage in patients with CNV, we evaluated the potential of 3K3A-APC treatment in halting CNV-associated leakage. Furthermore, looking ahead toward clinical application, we determined whether FA could serve as a surrogate marker for in vivo evaluation of the efficacy of 3K3A-APC treatment. FA has been established as a reliable tool for assessing CNV in human and murine models [2,42]. Therefore, as observed in patients with nAMD, we confirmed the presence of leakage from CNV and showed the efficacy of 3K3A-APC treatment by FA. Our results demonstrate that 3K3A-APC effectively inhibits leakage from clinically active CNV, with a statistically significant increase in the odds of forming non-leaking lesions by 20.43 (95% CI 2.19–190.50, *p =* 0.008) with 3K3A-APC treatment (Figure 5B). The current results are consistent with wt-APC-induced suppression of CNV leakage [31]. Additionally, we found significantly higher levels of VEGF in leaking CNV lesions than in non-leaking lesions. Previous reports have also shown a significant reduction in VEGF levels at the site of CNV induced by 3K3A-APC treatment and a longitudinal decrease in VEGF levels at the CNV area up to 14 days after treatment with wt-APC [18]. VEGF secretion has been identified in multiple cell types in the retina, including RPE cells, macrophages, microglia, astrocytes, and choroidal endothelial cells [3,5]. Nevertheless, the exact mechanism of how 3K3A-APC reduces VEGF levels in the retina and the cellular origins of VEGF is still unclear.

Moreover, we showed the efficacy of 3K3A-APC treatment in reducing CNV volume and invasion from the choroid toward the RPE (Figure 4B,C [18,31]). The RPE layer acts as an outer BRB that protects the sensory retina from excessive leakage from the fenestrated blood vessels of the choroid [6,43], and it lacks blood vessels in the healthy retina. Deterioration of the RPE and the BRB is an important pathological occurrence in the formation of CNV [3]. Wt-APC effectively seals the RPE blood barrier and reduces leakage towards the RPE by relocating the tight junction protein zonula occludens 1 (ZO1) to the RPE membrane [31]. Thus, the ability of 3K3A-APC to reduce the proximity of the leaky CNV to the RPE, along with its tightening effect on the RPE, may be a crucial step in protecting the retina from uncontrolled leakage and preventing damage to the sensory retina, providing another important potential target for the beneficial effects of 3K3A-APC.

The ITV route of administration is well-established and extensively utilized for delivering therapeutics to treat various retinal conditions in clinical practice. The selection of ITV administration in the current study was predicated on our previous research, which demonstrated its efficacy in murine models [18,19]. Furthermore, a clinical study substantiated that ITV administration of wt-APC is efficacious, reinforcing our rationale for choosing this route of administration [32]. However, safety concerns, potential complications, and patient comfort are important factors to be considered. With the aim of extending potential future applications in clinical practice, we conducted a preliminary experiment using the CNV murine model, involving intravenous administration of 3K3A-APC at a dosage of 0.2µg/kg. This dose is frequently used in rodent preclinical experiments focusing on neurodegenerative brain diseases [16,39,41]. Our initial results demonstrated that intravenous administration of 3K3A-APC resulted in a reduction in CNV depth and a decrease in CD11b+ cells accumulation. While these findings are preliminary and require further investigation, they suggest that systemic treatment with 3K3A-APC may also be a promising therapeutic approach for patients with nAMD.

The receptors EPCR, PAR1, and PAR3 have been identified as essential for the cellular activities of 3K3A-APC in the CNS [11,15]. Given that the retina is a part of the CNS and shares developmental origins [44], it is appealing to hypothesize that EPCR, which is strongly expressed in human retinal endothelial cells [45], and PAR1, which has been reported to be expressed in retinal ganglion cells, Müller glial cells, rods, and the inner nuclear layer [46,47,48], may mediate the signaling of 3K3A-APC in the retina. Furthermore, APC can act as a direct agonist of the Tie2 receptor and mimic the activity of its ligand angiopoietin 1 (Ang1) independently of EPCR or PAR1 [49]. We previously demonstrated that signaling via the Tie2 receptor is involved in the APC-induced regression of CNV and stabilization of the RPE [31]. Tie2 and Ang1 have been recognized as promising targets for the treatment of nAMD due to their roles in regulating angiogenesis and vascular stabilization [29,50] Thus, APC signaling through Tie2 may represent another potential target for the multi-target activities of APC in the retina, leading to CNV suppression.

In conclusion, our findings demonstrate that the anti-inflammatory activities of 3K3A-APC were accompanied by a regression of CNV growth and leakage. The quest for effective treatments to prevent visual loss from chronic progressive retinopathies necessitates the development of robust neuroprotective strategies. These strategies have the potential to halt photoreceptor death, thus averting subsequent vision loss [30,51]. Drawing insights from the neuroprotective activities of 3K3A-APC in the brain and CNS [11,15,16,17] and recognizing the retina, particularly the macula, as an extension of the brain, we propose 3K3A-APC as a potential neuroprotective treatment for AMD and other neurodegenerative retinal pathologies. Future studies are needed to evaluate the long-term neuroprotective effects of 3K3A-APC and to pave the way for clinical trials.

## 4. Materials and Methods

### 4.1. In Vivo Laser-Induced CNV Animal Model and Intravitreal Injections

The study included 8-week-old male C57BL/6J mice weighing 19 to 25 g purchased from Envigo RMS, Jerusalem, Israel (cat#BL-606). All animal experiments were performed according to the ARVO statement’s guidelines for the Use of Animals in Ophthalmic and Visual Research and the approval of the Institutional Animal Care and Use Committee at Rabin Medical Center. CNV was induced based on Weinberger et al. as previously described [20]. Briefly, a diode laser indirect ophthalmoscope (Iris Medical Oculight SLX System©, Iridex, Mountain View, CA, USA) was used with a laser power of 350 mW for a duration of 100 msec, and a condensing lens of 90 diopters. Two laser applications were applied to the right eyes, at a distance of 1 to 2 optic disc diameters around the optic nerve. Disruption of the Bruch’s membrane was identified by the appearance of a white bubble at the site of photocoagulation.

Mice were randomized to receive intravitreal (ITV) injection of either vehicle or 3K3A-APC.The ITV route of administration was selected based on our previous research on 3K3A-APC’s efficacy in ocular murine models, which consistently provided reliable and robust results [18,19].

Murine recombinant 3K3A-APC [KKK192-194AAA] was prepared as previously described [14]. Intravitreal 3K3A-APC concentration of 1 µg/µL/eye was chosen based on previously performed dose-dependent analysis of intravitreal injection of wt-APC, and a following study comparing ITV injection of wt-APC and 3K3A-APC [18,31]. According to standard dose conversion methods, specifically the body surface area normalization method, the estimated human equivalent dose would be approximately 0.00405 mg/kg [52]. Notably, safety studies of ITV administration of wt-APC in rabbit eyes demonstrated that a dose of ≤15 μg/eye was found to be safe, while 150 μg/eye was potentially toxic [53]. With the Km factor for mice being 3 and for rabbits being 12 [52], the estimated rabbit equivalent dose would be approximately 25 µg/rabbit, which suggests its safety.

Animals were anesthetized with intraperitoneal (IP) injection of ketamine 100 mg/kg and xylazine 10 mg/kg. 3K3A-APC was injected intravitreally under an operating microscope (Zeiss Opmi 6S Microscope; Carl Zeiss Microscopy GmbH, Oberkochen Germany), using a microsyringe (33-gauge; cat# 7803-05 Hamilton, OH, USA). Mice injected with vehicle (50% glycerin diluted in saline), with or without laser application, served as controls. In preliminary experiments, an additional control group of normal eyes injected with 3K3A-APC was compared to normal eyes injected with vehicle. Our findings showed no significant differences between these two groups in terms of the inflammatory markers measured, including IL-1β, NLRP3, and IBA+ cell counts (*p* = 0.8, 0.4, and 0.99, respectively). Therefore, we chose to consistently use the healthy control + vehicle group in our subsequent experiments. For all animal experiments, animal allocation to treatments was randomized.

### 4.2. Fluorescein Angiography (FA)

A total of 23 animals participated these experiments, 9 mice for the CNV group and 7 mice for each CNV+3K3A-APC or control group. After the mice were anesthetized, their pupils were dilated using tropicamide 0.5% (Fischer Pharmaceutical Labs, cat# 181010322, Bnei Brak, Israel), and 0.1 mL 2.5% fluorescein sodium (Serb Pharmaceutical cat #5042705, Paris, France) was injected IP. Sequential real-time photos were captured during the early phase (during the first minute from fluorescein injection) and late phase (every minute between 2 to 5 min following fluorescein injection). Color fundus photographs and FA images were taken using the Optos California UWF imaging system (Optos Inc., Dunfermline, Scotland). Two masked retina specialists evaluated the fluorescein angiograms and sorted each laser spot with “leakage,” when hyperfluorescent lesions showed blurred margins increasing in size over time and no leakage or “scar” when hyperfluorescent lesions showed distinct margins with no blur of margins over time.

### 4.3. Vascular Imaging and Flat-Mount Immunostaining

Mice were anesthetized 4 and 13 days post-treatment, and 0.1 mL of 25mg/mL fluorescein isothiocyanate (FITC) dextran conjugate (MW 500k, Sigma-Aldrich, cat#4697-500MG-F, St. Louis, MO, USA) was injected into the left ventricle of the mouse heart. The mice were sacrificed 5 min later, and a flat-mount specimen of the choroid was separated from the eyecup. Flat-mount specimens were fixed in 4% paraformaldehyde (PFA) for 10 min. Slides were incubated in phosphate-buffered saline (PBS)-Triton X100 0.5% solution at 4 °C overnight and later blocked for 2 h at room temperature (RT) in 5% normal donkey serum (NDS; Sigma-Aldrich, cat#4697-500MG-F, Rechovot, Israel). Slides were incubated overnight at 4 °C with the first antibody diluted in blocker: rat anti-mouse CD11b (1:200; Abcam cat #ab8878, UK), rabbit anti-mouse IBA1 (1:100 WAKO cat# 019-19741, Japan) and rabbit anti-mouse VEGFA (1:400; Abcam cat# ab46154, UK). The secondary antibody was incubated at 4 °C overnight; Alexa Fluor 568 conjugated goat anti-rat IgG, Alexa Fluor 568 conjugated goat anti-rabbit IgG, respectively (1:100; Invitrogen cat# A11077/A21206, Waltham, MA, USA). The slides were covered with an anti-fade reagent (Invitrogen cat# p36930 MA, USA). Negative controls were performed to confirm the specific binding of the antibodies by omitting the primary antibodies from the procedure. A specimen incubated with non-immune serum was used as a staining control. A specimen incubated with non-immune serum was used as a staining control. A total of 46 animals participated in these experiments: for IBA1 staining 23 mice, 10 mice for the CNV group and 7 mice for each CNV+3K3A-APC or control group.For CD11b and FITC-Dextran staining, 23 mice were used: 10 mice for the CNV group, 8 mice for CNV+3K3A-APC, 5 mice for the control group.

Images of 3 dimensional (3D) projections were captured as previously described [31], using the Leica TCS SP8 confocal microscope (Leica Biosystems, Nussloch, Germany) and the volume and the depth of FITC dextran staining were measured using the Imaris x64 7.1.1 software version 8.4 (Oxford Instruments, High Wycombe, UK). For choroidal thickness measurement, the Z axis of two images (one spanning the optic nerve and the other at a distal end) were measured at the center of each image and averaged. The volume of CD11b, IBA1 and VEGF staining was measured using Imaris x64 7.1.1 software (Oxford Instruments, High Wycombe, UK). A CD11b cell count was performed using Imaris software version 8.4 modelling each CD11b cell to a sphere, and counting the sphere number.

### 4.4. Cryosection Histology and Immunofluorescence Staining

This experiment included a total of 17 mice: 7 mice for CNV group, 5 mice for the CNV +3K3A-APC group, and 5 mice for the control group, divided into 2 separate repetition experiments. The mice were sacrificed 4 days post-treatment, and the eyes were removed, punched with a 30 g needle, and fixed in 4% PFA for 2 h at RT. The eyes were washed with increasing concentrations of sucrose in PBS and incubated with a final concentration of 30% sucrose overnight at 4 °C. The eyes were then embedded in OCT compound (Sakura Finetek cat#4583, Japan) on dry ice and kept at −80 °C. Serial sections of 10 µm thickness were cut using a cryostat (Leica Biosystems, Nussloch, Germany). Sequential cryosections of each eye from the lesion area were blocked with 3% NDS for 2 h at RT and then incubated with rabbit anti-mouse IBA1 (FujiFilmcat# 019-19741, WAKO, Japan) (2 μg), rabbit anti-human IL-1β (1:25, Abcam cat# ab9722, Cambridge, UK) or rabbit anti-mouse NLRP3 (1:200, Abcam cat# ab281559, Cambridge, UK) at 4 °C overnight. Slides incubated with non-immune serum were used as a staining control. The next day, sections were incubated with Alexa Fluor 488 conjugated donkey anti-rabbit secondary antibody (1:100, Invitrogen cat# A21206, USA). Nuclei were counterstained with DAPI (Nucblue fixed cell stain, MolecularProbes Invitrogene cat# R37606, MA, USA). Negative controls were performed to confirm the specific binding of the antibodies by omitting the primary antibodies from the procedure.

Images were captured using a fluorescence microscope (Nikon AX confocal system, Nikon, Tokyo, Japan) under the same conditions. The area of staining was measured using ImageJwin 64 version 10 software (NIH, Bethesda, MD, USA).

### 4.5. Statistical Analysis

Statistical analysis was performed using SPSS version 26 (IBM Corp., Armonk, NY, USA). Continuous variables were presented as the median and interquartile range (IQR). Categorical variables were presented as counts, proportions, and/or percentages. Because normality tests have little power to reject the null hypothesis that the data come from a normal distribution if sample size is small [54,55], the data were analyzed using non-parametric tests, which do not assume the data have any particular distribution. To evaluate the effect of 3K3A-APC treatment on NLRP3 and IL-1β levels, the number of IBA+ or CD11b+ cells at the CNV sites, the total CNV volume, and the penetration depth of blood vessels, we used the Kruskal–Wallis test followed by Dunn’s post hoc test. To evaluate the effect of 3K3A-APC on the laser lesion status taking into consideration that there were two laser lesions per mouse, we used a generalized estimating equation, with the lesion status (“leaking” or “non-leaking”) as a binary outcome and 3K3A-APC treatment (“no” or “yes”) as a between-subjects factor. To evaluate the effect of laser lesion status on VEGF volume taking into consideration that there were two laser lesions per mouse, a linear mixed model was used, with the VEGF volume as a continuous dependent variable and the lesion status (“leaking” or “non-leaking”) as an independent variable. Differences were considered statistically significant if the *p* value was less than 0.05.

## 5. Conclusions

Our findings demonstrate that the anti-inflammatory activities of 3K3A-APC were accompanied by a regression of CNV growth and leakage. Treatment with 3K3A-APC reduced the accumulation and activation of myeloid cells and microglia, as well as the levels of NLRP3 inflammasome and IL-1β at the CNV area. Given the complex and interconnected pathways involved in CNV development, the pleiotropic activities of 3K3A-APC offer a multi-target approach that may impede inflammation, defend BRB function, and protect the retina, thereby preserving vision. Furthermore, our data suggest that 3K3A-APC holds promise as a therapeutic option for the treatment of CNV in patients with nAMD, considering its established clinical safety profile in treating severe ischemic stroke [17]. Future studies are needed to evaluate the long-term neuroprotective effects of 3K3A-APC.

## Data Availability

Data are available from T.L. upon request.

## Supplementary Materials

The following supporting information can be downloaded at: https://www.mdpi.com/article/10.3390/ijms241310642/s1.
