# Supplementary material for: 3K3A-Activated Protein C Inhibits Choroidal Neovascularization Growth and Leakage and Reduces NLRP3 Inflammasome, IL-1β, and Inflammatory Cell Accumulation in the Retina
Source: Int J Mol Sci. 2023 Jun 26;24(13):10642. doi: 10.3390/ijms241310642 (PMC10341424; doi:10.3390/ijms241310642)
Supplement: Supplementary file 1 [file ijms-24-10642-s001.zip › ijms-2442752-supplementary.pdf]

Table S1. Effect of 3K3A-APC on the status of laser lesions estimated by a generalized estimating equation.

Dependent variables	Independent variables	B	SE of B	*P* value	Odds Ratio	95% CI for Odds Ratio	
Non-leaking lesion	Intercept	−0.45	0.55	0.412	0.64	0.22	1.87
	3K3A-APC treatment	3.02	1.14	0.008	20.43	2.19	190.50

Note: In the generalized estimating equation, the lesion status ("leaking" or "non-leaking") is a binary outcome and 3K3A-APC treatment ("no" or "yes") is a between-subjects factor. B, unstandardized coefficient; SE, standard error; CI, confidence interval; APC, activated protein C.

Table S2. Relationship between the lesion status ("leaking" or "non-leaking") and VEGF volume estimated by a linear mixed model with random intercept.

Dependent variables	Independent variables	B	SE of B	*P* value
VEGF volume	Intercept	4607	26781	0.865
	Leaking lesion	131529	47116	0.009

Note: B, unstandardized coefficient; SE, standard error; VEGF, vascular endothelial growth factor.
